# Exploring the Possible Role of Lysine Acetylation on *Entamoeba histolytica* Virulence: A Focus on the Dynamics of the Actin Cytoskeleton

**DOI:** 10.1155/2013/757392

**Published:** 2013-09-01

**Authors:** L. López-Contreras, V. I. Hernández-Ramírez, A. E. Lagunes-Guillén, Sarita Montaño, B. Chávez-Munguía, B. Sánchez-Ramírez, P. Talamás-Rohana

**Affiliations:** ^1^Departamento de Infectómica y Patogénesis Molecular, Centro de Investigación y de Estudios Avanzados del I.P.N., Avenida Instituto Politécnico Nacional No. 2508, Col. San Pedro Zacatenco, Del. Gustavo A. Madero, 07360 México, DF, Mexico; ^2^Laboratorio de Biotecnología, Facultad de Ciencias Químicas, Universidad Autónoma de Chihuahua, Apdo. Postal 1542-C, 31000 Chihuahua, CHIH, Mexico

## Abstract

Cytoskeleton remodeling can be regulated, among other mechanisms, by lysine acetylation. The role of acetylation on cytoskeletal and other proteins of *Entamoeba histolytica* has been poorly studied. Dynamic rearrangements of the actin cytoskeleton are crucial for amebic motility and capping formation, processes that may be effective means of evading the host immune response. Here we report the possible effect of acetylation on the actin cytoskeleton dynamics and *in vivo* virulence of *E. histolytica*. Using western blot, immunoprecipitation, microscopy assays, and *in silico* analysis, we show results that strongly suggest that the increase in Aspirin-induced cytoplasm proteins acetylation reduced cell movement and capping formation, likely as a consequence of alterations in the structuration of the actin cytoskeleton. Additionally, intrahepatic inoculation of Aspirin-treated trophozoites in hamsters resulted in severe impairment of the amebic virulence. Taken together, these results suggest an important role for lysine acetylation in amebic invasiveness and virulence.

## 1. Introduction

Lysine acetylation crucially modulates protein function and affects signaling pathways networks and thereby alters cell fate and function. Although lysine acetylation was initially related with regulation of nuclear transcription, however, more recently proteomic analyses have shown a large number of acetylated proteins in the cytoplasm, mitochondria, ER, and Golgi, including cytoskeletal proteins [1–4], suggesting that lysine acetylation might have the same relevance as phosphorylation in the biology of the cell. Lysine acetylation is involved in cytoskeleton remodeling, therefore affecting cell migration. In fact the cytoskeleton is indispensable for cell migration since it is essential for the formation of membrane ruffles or lamellipodia, filopodia, and actin stress fibers, which reflect different dynamic states of the actin cytoskeleton [5].

Cell migration is a highly dynamic phenomenon essential to a variety of biological processes such as morphogenesis, cancer metastasis, or parasite invasion [6–8]. The invasive process of *Entamoeba histolytica*, the etiologic agent of human amebiasis, is driven by motility, and cytoskeleton dynamics is indispensable for parasite invasion [9]. Moreover, it has been well documented that the actin cytoskeleton participates in capping formation in *E. histolytica, *which has been proposed as an effective way of evading the host immune response by rapid mobilization of surface receptors into the cap and its further release [10, 11]. 

Aspirin is a classic cyclooxygenase type I (COX-1) inhibitor, and irreversible inhibition of this enzyme is caused by acetylation of the COX-1 serine 530 residue. Recently it has been reported that Aspirin causes L-acetylation of cytoskeletal proteins, histones, heat shock proteins, and glycolytic and pentose phosphate pathway enzymes [12, 13]. Here, we explored the possible effect of protein acetylation in the behavior of *E. histolytica *trophozoites; we found that the increase of Aspirin-induced acetylation status of cytoplasmic proteins, particularly of actin, affected the cell cytoskeleton therefore reducing cell movement and capping formation and amebic virulence *in vivo*. These results show how Aspirin alters the acetylation state and function of proteins involved in virulence suggesting an important role for acetylation in eukaryotic parasite cells.

## 2. Materials and Methods

### 2.1. Cells

Trophozoites of *E. histolytica* HM1-IMSS strain were axenically cultivated in TYI-S-33 medium [14], supplemented with 10% (v/v) bovine serum and 3% (v/v) Diamond vitamin tween 80 solution (JRH Biosciences) for 48 h in glass screw cap tubes (16 × 125 mm) at 37°C. After that, cells were incubated on ice for 10 min, collected by centrifugation at 1100 rpm for 10 min, and washed three times in TYI-S-33 medium without serum. These trophozoites were then treated separately with the following drugs: 1 mM Aspirin, 50 *μ*m Indomethacin (Indo), 50 *μ*m NS-398 or 10 *μ*m Cytochalasin D (CD), all of them diluted in 1% DMSO, for 2 h at 37°C, and then they were washed with TYI-S-33 medium without serum. Trophozoites (1.5 × 10^6^) nontreated or treated with Aspirin were used to determine lysine acetylation, rearrangement of the actin cytoskeleton, and amebic movement; amebic liver abscess (ALA) development in the hamster model was induced by inoculation of nontreated trophozoites (control, *n* = 9) or trophozoites treated with Aspirin (*n* = 9), Indo (*n* = 9), or CD (*n* = 5).

### 2.2. Western Blot and Immunoprecipitation

Total cell lysates from equivalent cell numbers of each tested condition were resolved by 10% SDS-PAGE. Proteins were then transferred onto nitrocellulose membranes (Bio-Rad, Hercules, CA). Membranes were blocked with 5% nonfat dry milk in Tris Buffered Saline (TBS) for 2 h at room temperature. Membranes were probed overnight at 4°C with antiacetyl-lysine antibody (1/200) (Cell Signaling Technology Inc.) in TBS. Membranes were washed with TBS-Tween 5% and then incubated with goat anti-rabbit IgG conjugated to horseradish peroxidase (1/1000) for 2 h at room temperature. After washing with TBS-Tween 5%, antibody-reactive proteins were detected by chemiluminescence, using the substrate Super Signal (Pierce, Rockford, IL) according to the manufacturer's instructions.

For immunoprecipitation, cell lysates (1 mg of total protein) were precleared with protein G-agarose (Gibco-BRL, Grand Island, NY) (previously blocked with 2% bovine serum albumin) for 2 h at 4°C. The antiactin antibody (1/500) was then added to the cell lysates supernatant. Mixtures were incubated overnight at 4°C, and then 2% BSA blocked protein G-agarose was added and incubated for another 2 h at 4°C. Agarose beads were recovered by centrifugation at 11,600 ×g for 2 min at 4°C, washed with 10 mM Tris-HCl pH 7.4, containing 150 mM NaCl, 3 mM EDTA, and 1% Nonidet P-40, resuspended in Laemmli's sample buffer, and boiled for 5 min. After centrifugation, supernatants were loaded onto a 10% SDS-PAGE and then processed as described previously with antiacetyl-lysine (1/200) and antiactin (1/1000) antibodies and their respective secondary antibodies.

### 2.3. Confocal Microscopy and Movement Analysis

Trophozoites treated or not with 1 mM Aspirin were fixed with 4% formaldehyde, blocked with BSA for 1 h at 37°C, and incubated overnight with the antiacetyl-lysine antibody (1/50) (Cell Signaling Technology). Then, cells were washed with PBS, incubated with FITC-labeled goat anti-rabbit IgG (1/100; Jackson ImmunoResearch; Pennsylvania, USA) secondary antibody. Actin was stained with rhodamine-phalloidin (1 : 25 Molecular Probes; Oregon, USA) for 30 min at 37°C. Coverslips, mounted with Vectashield (Vector Laboratories; Ontario, Canada), were analyzed by confocal microscopy in a Leica TSCNT microscope (Leica Lasertechnik GMB; Illinois, USA). Aspirin-treated or nontreated live trophozoites' movement was recorded in video movies (three independent experiments for each condition). Images were captured in the time series mode over periods of 2–5 min, all at the same speed (14 frames/second), and once the videos were taken, approximately 500 frames/sample were selected for further analysis. The movement of trophozoites (speed and distance traveled with respect to a reference point) was measured and analyzed using the track object feature of the Image-Pro plus V 5.1 software (Media Cybernetics, Inc., Maryland, USA). Motion dynamics were measured by making frame to frame comparisons between conditions.

### 2.4. Immunoelectron Microscopy

Trophozoites treated or not with 1 mM Aspirin were washed three times with PBS and once with serum-free DMEM. Cells were fixed in 4% paraformaldehyde and 0.1% glutaraldehyde in serum-free DMEM for 1 h at room temperature. Samples were embedded in LR-White and polymerized under UV at 4°C overnight for 48 h. Sections were obtained and mounted on formvar-covered nickel grids. Later they were incubated with antiactin and antiacetyl-lysine antibodies (1 : 40). Actin was detected with 15 nm colloidal-gold-coupled anti-mouse (1 : 40) and acetylated lysine with 30 nm colloidal-gold-coupled anti-rabbit antibody (1 : 40). Thin sections were observed with a Jeol JEM-1011 transmission electron microscope.

### 2.5. Actin Sequences Alignment

Actin sequences from *Homo sapiens* (Accession NP_001091.1), *Mus musculus* (Accession AAA37164.1), and *Entamoeba histolytica* (AAA29086) were aligned using ClustalW2 (http://www.ebi.ac.uk/Tools/msa/clustalw2/).

### 2.6. Template Search of Actin from *Entamoeba histolytica*

The amino acid sequence of actin of *Entamoeba histolytica* was retrieved from the sequence database of NCBI (http://www.ncbi.nlm.nih.gov/) (locus:XP_001913786). The three-dimensional structure of the protein actin was not yet available in Protein Data Bank (PDB) [15]; hence to build a 3D molecular model of *E. histolytica* actin, an alignment in BLASTP [16] with the data base from PDB with default parameters was performed to find suitable templates from homology modeling. Based on the maximum identity with a high score (99%) and lower *e*-value (0.0) crystal structure of actin (PDB code:4EFH) of *Acanthamoeba castellanii* was taken as a template [17].

### 2.7. 3D Structure Generation

The 3D molecular model was built with I-TASSER server (http://zhanglab.ccmb.med.umich.edu/I-TASSER) [18] using as a template actin from *Acanthamoeba castellanii* (4EFH:A). The C-score between the query and the protein template and TM score estimated were 1.88 and 0.98, respectively.

### 2.8. Docking Studies

Molecular docking studies were performed to illustrate the probable binding site of Aspirin to *E. histolytica *actin. The geometry optimization of ligand Aspirin was done with Gaussian 03 [19] utilizing AM1 base. The Lamarckian genetic algorithm was selected for the ligand conformational search. The docking area was defined using Autogrid4. The grid options, number of points in *X* dimension, *Y* dimension, and *Z* dimension were 126 of each one, respectively, and the grid maps had a spacing of 0.375 $\overset{´}{Å}$. Additional docking parameters were number of GA runs: 100, populations size: 100, maximum numbers of evaluations: 10000000, maximum number of generation: 27000, rate of gene mutation: 0.02, Rate of crossover: 0.8, and number of generation for picking: 100.

### 2.9. Capping Induction

Incubation of live trophozoites of *E. histolytica* with FITC-concanavalin A (Con A) was carried out as described earlier [10, 11]. After 15 min of interaction with the lectin, cells were fixed with 4% paraformaldehyde for 30 min, and actin was revealed with rhodamine-phalloidin (1 : 25) and nucleus with DAPI (1 : 300). Finally, samples were observed by confocal microscopy in a Leica TSCNT microscope (Leica Lasertechnik GMB; Illinois, USA). 

### 2.10. Amebic Liver Abscess Formation

Male hamsters *(Mesocricetus auratus)* of approximately 80–100 g were intrahepatically infected [20] with 1.5 × 10^6^ drug-treated or nontreated trophozoites (as described previously). Seven days after infection, animals were anesthetized with sodium pentobarbital (94.5 mg/kg of body weight) and killed by exsanguination. Livers were dissected and weighed before and after removing amoebic abscesses. To determine the percentage of hepatic damage abscesses were dissected from livers, and the mass of the abscess was divided by the mass of the whole liver and multiplied by 100. 

### 2.11. Statistical Analysis

Significant differences in cell movement between control and treated cells and significant differences in abscesses development between animals infected with parasites treated or not with different drugs were statistically analyzed by paired Student's *t*-test (*P* < 0.05). All statistical analyses were carried out using Stata software version 17.0.

## 3. Results and Discussion 

### 3.1. Aspirin Induces Actin Acetylation in *Entamoeba histolytica *without Affecting the Growth of the Parasite

**Figure 1:**
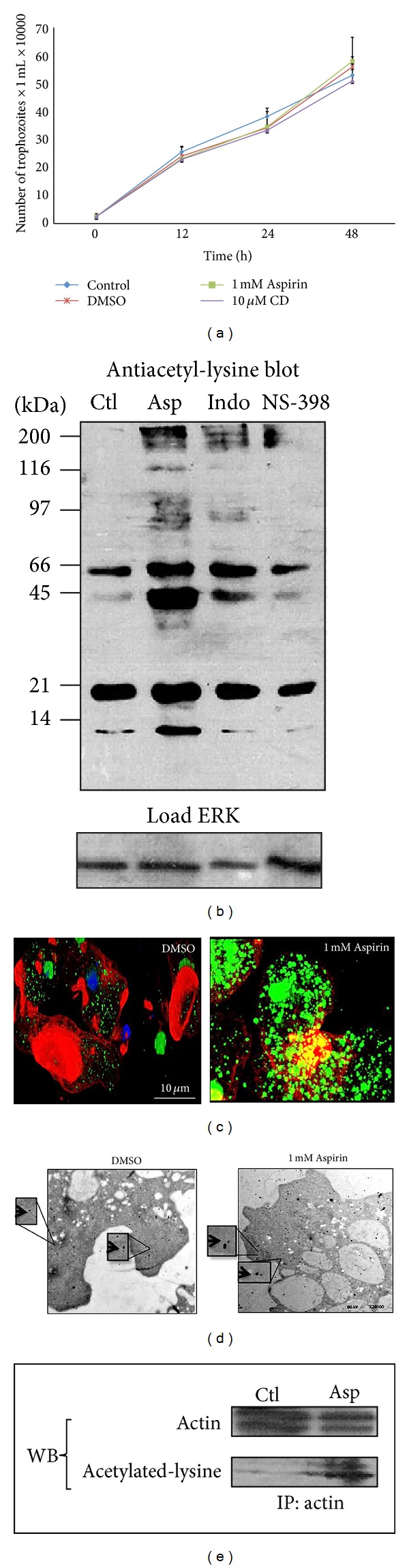
Aspirin acetylates actin in *E. histolytica *without affecting the growth of parasites. (a) Growth curves for untreated and Aspirin (1 mM), CD (10 *μ*M), or DMSO-treated *E. histolytica.* (b) Trophozoites were treated with 1 mM Aspirin, 50 *μ*m Indo, or 50 *μ*m NS-398 for 2 h, lysates prepared and immunoblotted with antiacetyl-lysine antibody (1 : 200). (c) Confocal microscopy analysis of trophozoites treated or not with 1 mM Aspirin. Actin was stained with rhodamine-phalloidin (red) (1 : 25), and acetylated proteins (green) were detected with antiacetyl-lysine antibody (1 : 50) and FITC-labeled goat anti-rabbit IgG (1 : 100). (d) Transmission electron micrographs show a section of trophozoites treated or not with 1 mM Aspirin, where the association between gold-labeled acetyl-lysine antibody (large particles) and actin (small particles) (arrows) is clear. (e) Detection by western blot of the acetylated level of immunoprecipitated proteins from treated (Asp) and nontreated (Ctl) trophozoites with the antiacetyl-lysine antibody (1 : 200).

To exclude a toxic effect by 1 mM Aspirin on trophozoites' viability, cell viability was determined at the end of the treatment period, using the Trypan blue exclusion test, finding that in all experiments cell viability was >90%. In addition, an aliquot of drug-treated or nontreated trophozoites was set up for a growth curve; after 48 h, cells in all conditions had grown with a similar rate, confirming that these treatments (Aspirin, CD or DMSO) did not have an effect on the growth of trophozoites (Figure 1(a)).

To demonstrate that trophozoites treatment with 1 mM Aspirin for 2 h induced protein acetylation in this parasite, lysine acetylation of proteins was shown by immunoblotting (Figure 1(b)). Only a limited number of acetylated proteins were detected in the untreated control or in trophozoites treated with other COX inhibitors (Indo or NS-398); however, Aspirin increased both the number and the level of acetylation of some proteins. In particular, acetylated proteins with apparent molecular weight of 66, 45, 21 and 14 kDa were seen (Figure 1(b)); confocal microscopy confirmed the acetylation of cytoplasmic and nuclear proteins in Aspirin-treated trophozoites (Figure 1(c)). The finding of lysine acetylation of a 45 kDa protein (Figure 1(b)) and colocalization of F-actin and acetylated proteins by confocal microscopy (Figure 1(c)) suggested that actin (42 kDa) could be a substrate for Aspirin acetylation in trophozoites. Therefore, we immunoprecipitated actin in Aspirin-treated trophozoites and detected lysine acetylation by western blot (Figure 1(e)). Moreover, results by MET showed that acetylated actin is present in actin-rich structures formed in trophozoites (Figure 1(d)). 

It has been reported that Aspirin acetylates several cellular proteins in cancer cells [12, 13]. In this case, besides the presence of physiological lysine acetylation, Aspirin induced a noticeable increase in the amount and levels of acetylated proteins. 

### 3.2. Docking Studies Suggest That Aspirin Acetylates Lys 62 and Affects the Actin Cytoskeleton Dynamics by Preventing Actin Polymerization

**Figure 2:**
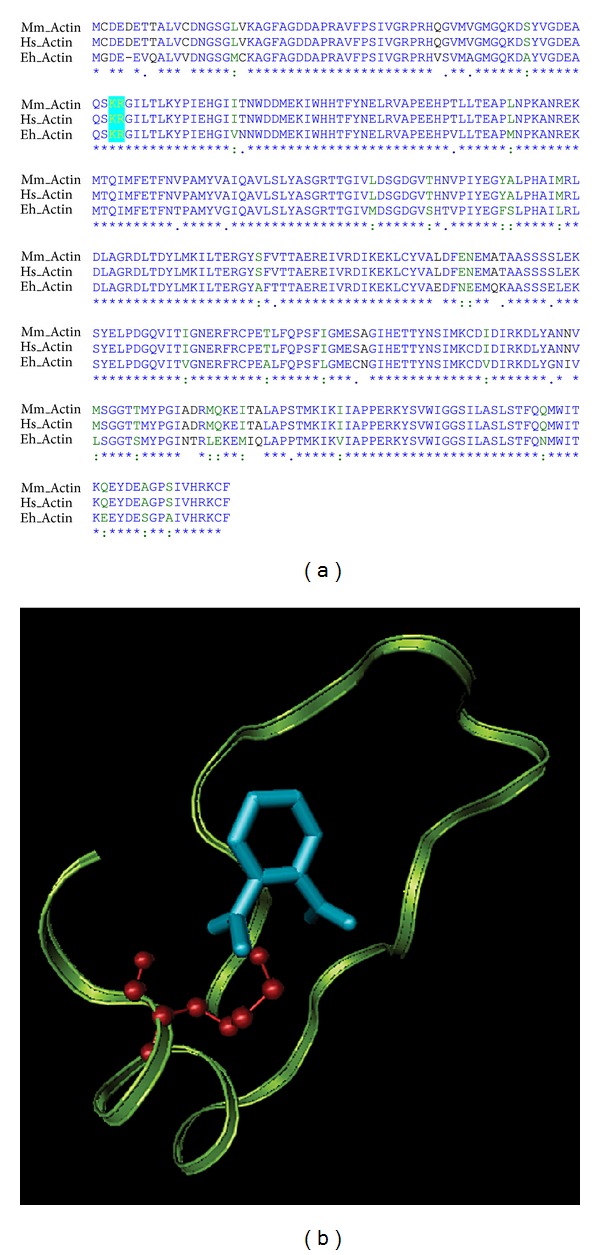
Predicted binding site of acetylsalicylic acid in actin from *Entamoeba histolytica*. (a) Alignment of actin from *Homo sapiens, Mus musculus*, and *Entamoeba histolytica*. Identical amino acid residues are in blue and similar amino acid residues in green; in blue box are lysines and arginines, important residues for actin polymerization. (b) Amino acids from 37 to 64 are explicitly shown, and the actin around acetylsalicylic is depicted in blue and lys 62 in red.

**Figure 3:**
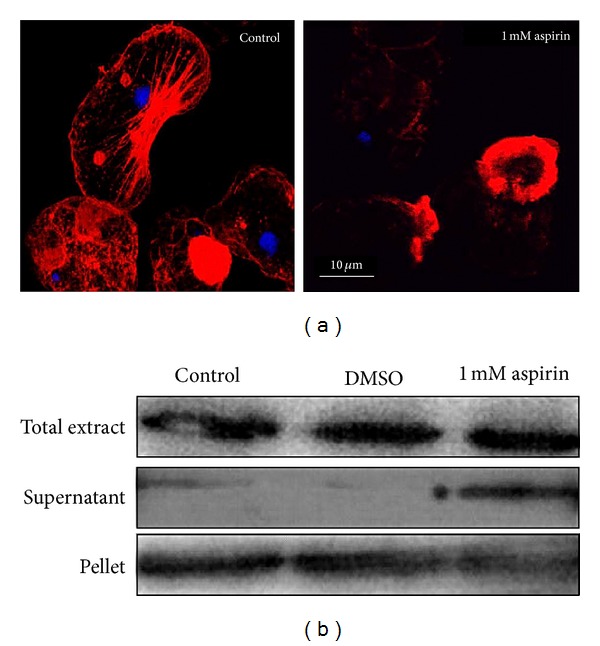
Aspirin affects the dynamics of the actin cytoskeleton and its polymerization in live trophozoites. (a) Confocal microscopy analysis of trophozoites treated or not with 1 mM Aspirin. Actin was stained with rhodamine-phalloidin (red) (1 : 25) and nucleus with DAPI (blue) (1 : 300). (b) FN-adhered trophozoites, treated or not with Aspirin for 2 h, were extracted with 0.5% Triton X-100 and separated into 8,000 ×g supernatant and pellet fractions, where actin was detected by western blot with antiactin antibody (1 : 5000).

Using proteomic approaches it has been shown that actin can be acetylated at lysine 61 [3]. This is important, because lysine 61 is a residue immediately adjacent to a critical arginine required for actin polymerization [21]. *In silico* analysis showed that both amino acids are present in *E. histolytica* actin sequence; however, these positions are shifted one place, lysine is 62 and arginine 63, even though the actin sequence is highly conserved (Figure 2(a)). Moreover, docking studies showed that acetylsalicylic acid from Aspirin binds at a distance of 2.55 Å to lysine 62 on actin from *E. histolytica* with Δ*G* = −5.12 and *K*_*d*_ =  175.93 *μ*m (Figure 2(b)). These results suggest that Aspirin could affect actin polymerization in *E. histolytica*. To confirm this hypothesis, FN-adhered trophozoites [22], treated or not with Aspirin for 2 h, were lysed with 0.5% Triton X-100, and proteins were separated into 8,000 ×g supernatant and pellet fractions. Under these conditions, G-actin is found in the supernatant fraction and polymerized actin (F-actin) in the pellet.  Figure 3(b) shows that in trophozoites treated with Aspirin, actin was present both in the supernatant and the pellet, while in untreated or DMSO controls, actin was found in the pellet. This experiment indicates that in live trophozoites, Aspirin affects actin polymerization during adhesion to FN, an extracellular matrix protein known to induce actin polymerization in eukaryotic cells and in amebas [22, 23].

Moreover, it has been reported that in highly invasive prostate cancer cells, actin cytoskeleton rearrangement and consequently cell adhesion and cell motility are affected by Aspirin [24]. The effect of an increased acetylation status on the actin cytoskeleton rearrangement was analyzed by confocal microscopy. *E. histolytica* actin cytoskeleton undergoes dynamic rearrangements and forms diverse actin structures such as phagocytic invaginations, adhesion plates, actin dots in association with adhesion plates and stress fibers, and pseudopodia [22].  Figure 3(a) shows that in control trophozoites, most of these structures were present, while in treated trophozoites, Aspirin produced alterations on the actin cytoskeleton rearrangement, and very few cytoskeleton structures were found. Control cells treated with DMSO or other COX inhibitors (Indo or NS-398) did not show affectation on the actin cytoskeleton rearrangement (data not shown), indicating that Aspirin specifically disturbed actin cytoskeleton structuration by impairing actin polymerization.

### 3.3. Reduction of Amebic Movement and Capping Formation in *E. histolytica* Might Be a Consequence of Actin Acetylation

**Figure 4:**
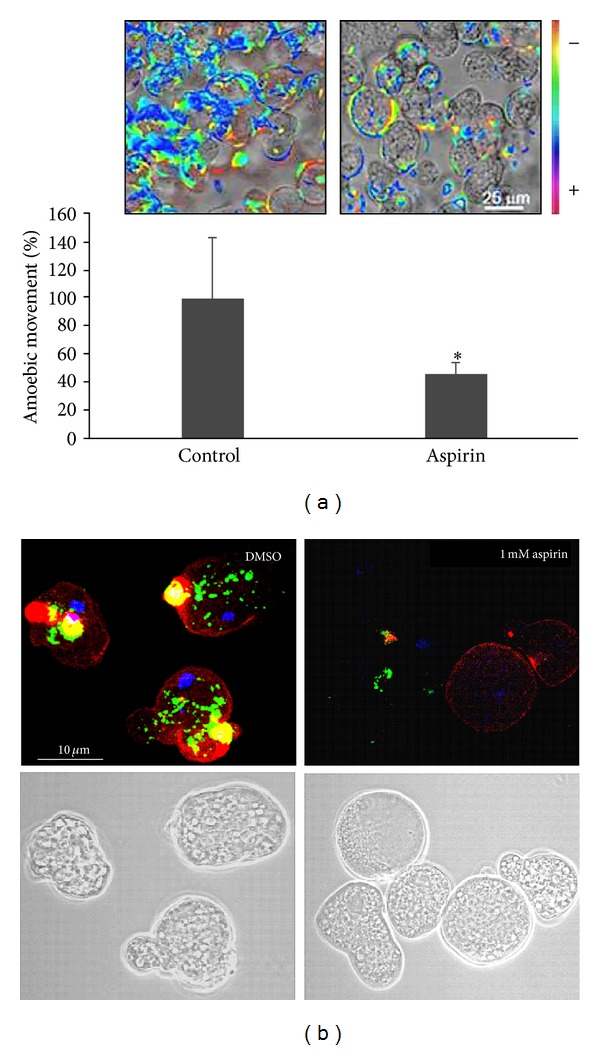
Aspirin decreases amebic movement and capping process. (a) The movement of live trophozoites treated or not with 1 mM Aspirin was evaluated as described in Section 2; colors for tracks were generated randomly, so every track has different color. Results are representative of three independent experiments, and the analysis represents the comparison of 500 frames. (b) Capping produced in *E. histolytica* trophozoites following interaction with ConA. Actin was stained with rhodamine-phalloidin (red) (1 : 25) and nucleus with DAPI (blue) (1 : 300); ConA is coupled to FICT (green).

Ameboid movement requires an intact and functional cytoskeleton; therefore, amebic movement was analyzed in Aspirin-treated trophozoites. Results suggest that the increase on actin acetylation by Aspirin diminishes amebic movement (Figure 4(a)). The effect of DMSO on the amebic movement was tested also, finding that DMSO did not affect this feature (data not shown). This result is in agreement with a previous report in which Aspirin diminished cell motility in highly invasive prostate cancer cells [24]. Also the movement of the parasite *Setaria cervi* is diminished by Aspirin [25], although this report does not explain the mechanism of movement reduction by Aspirin; our results might contribute to understand this phenomenon in *Setaria cervi*.

Capping formation is another actin cytoskeleton dependent process [10, 11]. With this purpose we examined, by confocal microscopy, the mobilization of Con A towards the posterior cap in trophozoites treated or not with Aspirin or with DMSO, after interaction with Con A for 15 min. Trophozoites treated with Aspirin did not show well-defined caps, while trophozoites treated with DMSO showed caps rich in actin as well as colocalization with Con A (Figure 4(b)). This result suggests that parasites treated with Aspirin are less efficient in capping formation because Aspirin may be affecting actin polymerization and therefore those functions that depend on the rearrangement of the cytoskeleton.

### 3.4. Amoebic Virulence Impairment Might Be due to Increased Level of Proteins Acetylation, Particularly Actin

**Figure 5:**
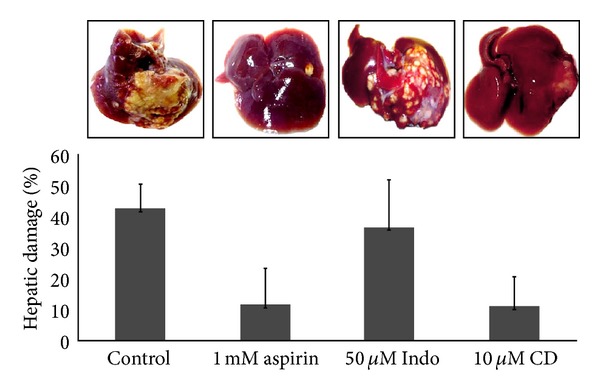
Aspirin and CD treatment of trophozoites reduce amebic virulence *in vivo*. Liver abscesses developed after 7 days after infection with live trophozoites of *Entamoeba histolytica* treated or not for 2 h with 1 mM Aspirin, 50 *μ*m Indo, or 10 *μ*m CD. The graphic shows the average ± S.D. (*n* = 9, except for CD where *n* = 5). Data are from three independent experiments. *P < 0.05.

The main marker of *E. histolytica* virulence is its capacity to produce liver abscesses in hamsters; this model is used for the study of hepatic human amebiasis because it is a susceptible model and mimics the human disease [26]. Thereby we decided to evaluate the effect of hyperacetylation, produced by Aspirin, on amebic liver abscess development.  Figure 5 shows that Aspirin-treated parasites had a notoriously diminished capacity for amebic abscesses development compared with nontreated or Indo-treated parasites. This result could possibly be explained by the fact that Aspirin reduces motility and capping formation of trophozoites, functions that have been associated with the pathogenesis of *E. histolytica *[9]. It has been shown that noninvasive *E. dispar* lacks the capacity for displacement and elimination of surface antigen-antibody complexes [27]; for this reason *E. dispar* is susceptible to the humoral immune response of the host. Our results are in agreement with this observation, because when amebas failed to move and to form caps, they also failed to form amebic liver abscesses; these results could suggest that amebas treated with Aspirin could have been eliminated by the humoral immune response.

To confirm that the reduction in amoebic virulence was largely due to the impairment of the actin cytoskeleton by the acetylation process, an additional experiment was conducted, in which hamsters were inoculated with 10 *μ*M cytochalasin D (CD)-treated trophozoites, a drug that has been shown to affect *E. histolytica* pathogenesis [28, 29]; this drug binds to actin filaments and blocks polymerization and fibers elongation. As with Aspirin-treated trophozoites, CD-treated trophozoites had a reduced capacity to produce amebic liver abscess (average inhibition of liver damage: 80%). Thus, these results strongly suggest that when affecting those functions that depend on the correct functioning of the actin cytoskeleton, there is also an impact on amoebic virulence.

It is important to note that other virulence factors, as was the case for erythrophagocytosis and proteolytic activity, were not affected by Aspirin treatment (data not shown).

Taken together, these results suggest a possible relevance for acetylation in amebic virulence and that Aspirin through acetylation of important cellular proteins, such as actin, might modulate amebic functions.

## 4. Conclusion

Information about the role of lysine acetylation of proteins is scarce, particularly in parasites. In this work, we have shown that amebic virulence is possibly modulated by acetylation status and also that Aspirin, which induces actin acetylation, alters the cytoskeleton structuration and function. Still, there are some other important proteins that could also be affected by acetylation, which is the case for histones. Our findings open up the way to investigate the role of acetylation on many vital cellular proteins.
